# Development of a 16-Channel Broadband Piezoelectric Micro Ultrasonic Transducer Array Probe for Pipeline Butt-Welded Defect Detection

**DOI:** 10.3390/s22197133

**Published:** 2022-09-21

**Authors:** Bolun Li, Changhe Sun, Shouchun Xin, Mingzhang Luo, Chuang Hei, Guofeng Du, Ankang Feng

**Affiliations:** 1 School of Electronics and Information, Yangtze University, Jingzhou 434023, China; 2 National Demonstration Center for Experimental Electrical & Electronic Education, Yangtze University, Jingzhou 434023, China; 3 School of Urban Construction, Yangtze University, Jingzhou 434023, China

**Keywords:** pipe butt weld, piezoelectric micro ultrasonic transducer array, impedance characterization, large bandwidth, defect detection

## Abstract

Butt welding is extensively applied in long-distance oil and gas pipelines, and it is of great significance to conduct non-destructive ultrasonic testing of girth welds in order to avoid leakage and safety accidents during pipeline production and operation. In view of the limitations of large transducer size, single fixed beam angle, low detection resolution and high cost of conventional ultrasonic inspection technologies, a 16-channel piezoelectric micro ultrasonic transducer (PMUT) array probe was developed through theoretical analysis and structural optimization design. After the probe impedance characterization, the experimental results show that the theoretical model can effectively guide the design of the ultrasonic transducer array, offering the maximum operating frequency deviation of less than 5%. The ultrasonic echo performance tests indicate that the average −6 dB bandwidth of the PMUT array probe can be up to 77.9%. In addition, the fabricated PMUT array probe has been used to successfully detect five common internal defects in pipeline girth welds. Due to the multiple micro array elements, flexible handling of each element, large bandwidth and high resolution of defect detection, the designed PMUT array probe can provide a good application potential in structural health monitoring and medical ultrasound imaging fields.

## 1. Introduction

Due to the advantages of large transportation volume, low cost and low dependence on the local-level climatic impact during operation, the pipeline has been widely adopted in the oil and gas transportation [1]. In the construction of long-distance pipelines, the girth butt welding is inevitably used, and its processing quality will directly determine the safety, reliability and service life of the entire engineering structures. Generally, most of the defects in the pipelines are formed near the welds, and the incidence rate will decrease as the distance from the weld increases [2]. During 2010–2019, there were 31 significant girth weld failures of onshore oil and gas transmission pipelines reported in the United States, and the total economic losses were up to USD 192 million [3]. Therefore, in order to avoid any leakage and safety accident caused by the weld defects, it is of great significance to conduct non-destructive testing (NDT) of pipelines during pipeline production and operation [4,5].

There are several existing NDT methods for pipelines, mainly including radiographic (e.g., X-ray, γ-ray) testing, magnetic particle testing, ultrasonic testing and eddy–current testing [6,7]. In general, radiographic testing has a low detection accuracy for cracks, incomplete fusion and other defects in the pipe girth butt welds. Furthermore, radioactive sources can cause irreversible harm to inspectors [8]. Although the magnetic particle testing and eddy–current testing methods are simple to operate and are not affected by the shape and size of the workpiece, they can only detect near-surface defects and cannot obtain pipeline internal information such as the depth of defects [9]. Instead, ultrasonic testing can detect various types of defects and has a good sensitivity to small defects. Furthermore, because of the advantages of small system size, easiness to carry and penetrate into the structures, and harmlessness to the human body, ultrasonic testing has become a promising on-line NDT technology [10,11].

At present, ultrasonic NDT technologies for weld defects can be mainly classified into: ultrasonic-guided wave detection, ultrasonic pulse-echo detection and ultrasonic phased-array testing. In the early 1990s, Nassar et al. proposed the finite element method (FEM) and the modal analysis method to study the reflected waves of axisymmetric-guided waves at the cracks in the welds of the plate [12]. Zhuang et al. used finite element analysis (FEA) to investigate the reflection characteristics of axisymmetric-guided waves by the cracks and weldments in the welded steel pipes [13]. Studies have shown that the reflection coefficients are sensitive to the length and slope of the crack, and the cracks dominate the scattering amplitudes, although the weldment will also affect the reflection characteristics markedly. However, they did not explicitly give a basis for judging defects in the weld area. Thus far, the welding defect detection of ultrasonic-guided waves is still challenging in real applications, although a handful of research studies have made some progress on the analysis of the weld feature from guided wave signals in the time and/or frequency domains [14,15,16,17]. Since the weld and the steel pipeline have some difference in the Young’s modulus and density, and because the multimodal and dispersion characteristics of the guided waves cannot be ignored, there are reflection waves in both the weld and the defects. Considering that these reflection waves are always superimposed, their waveforms are too complicated to analyze [10,18,19,20]. Moreover, the interpretation of the guided wave-based detection data is highly dependent on well-experienced practitioners, which may result in lower detection efficiency and higher cost [21].

Ultrasonic pulse-echo detection method is an alternative testing technology for evaluating the structural integrity of the girth butt-welded pipelines, which is based on analyzing the ultrasonic propagation path and the A-scan signals [22]. Unfortunately, the analysis based on the amplitude of signal reflected to receivers is always inconspicuous and unreachable owing to the lack of signal strength from a single probe [23]. Compared with the traditional single-element ultrasonic transducers, multi-element arrays, which perform a series of different azimuth checks from one location simultaneously, can offer great potential to improve inspection quality and reduce inspection time. Furthermore, the array-type transducers are more convenient and are efficient to use [24]. Early ultrasonic inspection systems that emerged in the 1990s placed multiple transducer probes in a probe tray to cover the entire weld area, with fixed transmitting ultrasonic beam direction and focusing depth [25]. In 2002, Song et al. used the PAULI ultrasonic system to evaluate the inside of nuclear power plant components via electronically scanned ultrasonic images, which improved the efficiency of flaw detection and localization compared to classical ultrasonic testing (UT) using the A-scan signals but failed to distinguish the defect categories from the images [26].

In order to further improve the detection efficiency and the flexibility of ultrasonic beams, the ultrasonic phased-array testing technologies have been paid with much attention. In 2009, Li et al. developed an ultrasonic phased array system for automatic flaw detection of the pipeline girth welds [27]. In 2016, Yamamoto et al. developed a phased-array ultrasonic inspection system (PAUT) with a 2 MHz, 128-element array probe, which can inspect cast austenitic stainless steel parts in nuclear power plants and distinguish defect echoes from the noises, offering slit sizing error of less than 2 mm [28]. In 2019, Fu et al. designed a 2.5 MHz 64-element binary matrix array probe and a 2.25 MHz 64-element single-wire array probe to detect defects in the stub butt welds of the China Fusion Engineering Testing Reactor (CFETR) vacuum vessel (VV) body and port, achieving width detection sensitivity of 0.2 mm [29]. However, ultrasonic phased-array probes have their own drawbacks, including complicated excitation and a reception system, limited operation bandwidth, low compatibility and high power consumption. Especially, the volume of the phased-array probe makes it impossible to scan the complete surface of components in complex structures with tight spaces. Even if the transducer can scan these parts, the ultrasonic beam cannot cover the target detection area due to limited scanning space [30]. In recent years, with the rapid development of microelectromechanical system (MEMS) technology, piezoelectric micro ultrasonic transducers (PMUTs) have attracted more and more attention due to small size, low power consumption, ease of matching with acoustic impedance and multi-element array design. Until now, PMU-based array probes have been successfully used in intravascular ultrasound (IVUS) imaging [31], B-scan medical imaging [32], fingerprint sensing [33,34] and ultrasonic radar ranging [35], which indicate its great potential in NDT-based microdefect detection.

In this work, a 16-channel broadband PMUT linear array probe with a custom 16-channel signal line connector is developed for pipeline butt-welded defect detection. All 16 PMUT elements are able to work independently in the A-scan/B-scan modes or work cooperatively in the phased-array imaging mode. Even if one or several elements fail to work, the other elements will not be affected. Such flexible handling of the PMUT array makes our design much more competitive than conventional phased-array probes. The necessary theoretical analysis has been performed to guide the structure design of the proposed PMUT linear array probe. Compared with the theoretically designed frequency, the measured frequency deviation of the fabricated probe is less than 5%. Both the electrical impedance characterization and ultrasonic pulse echo testing demonstrate this probe operating at about 5 MHz with the average −6 dB bandwidth of 77.9%, which is larger than most previously reported piezoelectric ultrasonic transducer arrays, especially PMUT arrays [36,37,38]. The developed PMUT array probe has been applied to successfully detect five common internal defects (gas pores, incomplete penetration, cracks, incomplete fusion and slag inclusion) in pipeline girth butt welds with a minimum feature size of less than 500 μm, exhibiting a high detection resolution. Such comprehensive demonstration for detecting various kinds of welded defects with the PMUT array is little studied. Benefited from its very simple and miniature structure and advantageous behaviors, the array probe has excellent application prospects in industrial non-destructive testing.

## 2. Design and Theoretical Analysis

**Figure 1:**
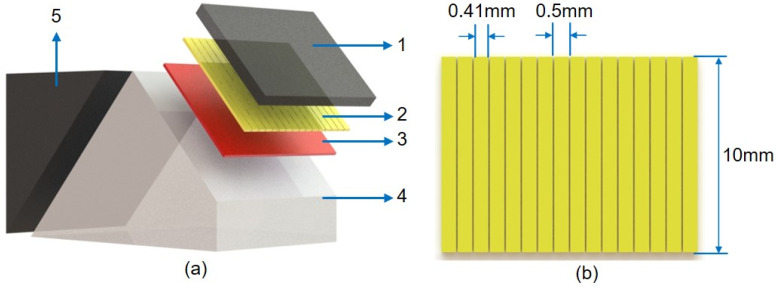
Structural design of 16-channel PMUT linear array probe: (**a**) exploded view, 1-backing damping block, 2-piezoelectric film linear array, 3-acoutic impedance matching layer, 4-wedge block, 5-sound absorbing block, (**b**) geometry of the piezoelectric film linear array.

The designed PMUT array probe is mainly composed of a metal-protecting shell, piezoelectric film linear array, backing damping block, acoustic impedance matching layer, wedge block and sound absorbing block, as shown in Figure 1. Piezoelectric films are selected from PZT-5H material with high piezoelectric constants and excellent electromechanical properties. All PZT-5H films are designed as rectangular strips, and each PZT-5H film is sandwiched between two silver thin films as electrodes, which are led out by signal transmission lines. The piezoelectric film linear array consists of 16 horizontally arranged piezoelectric elements with a length of 10 mm and pitch of 500 μm. The backing damping block adheres to the back and sides of the piezoelectric film linear array, and its material is a tungsten powder–resin–glass ball composite, which is mainly used as a fixed sensing element and absorbs vibrations on the back of the sensing element.

The adopted effective vibration mode of the piezoelectric films is based on the thickness expansion (TE) vibration so that its excited ultrasonic longitudinal waves will only propagate in the direction perpendicular to the surface of the piezoelectric film. In order to make ultrasonic waves transmitted into the steel pipe butt weld at a certain angle, a wedge block is selected to achieve waveform conversion according to the refraction principle. Furthermore, to eliminate the influence of multiple reflections and scattering echoes in the wedge block, a sound absorbing block that can absorb the echoes in the wedge block is also designed and embedded in the rear end face of the wedge block. The angle between the sound absorbing block and the piezoelectric elements is 90° in order to prevent the interference from the ultrasonic side lobes and the reflected echoes in the wedge block. The material of the sound absorbing block is a tungsten powder–epoxy resin composite. The acoustic impedance matching layer is one protective and ultrasonic coupling layer between the piezoelectric film linear array and the wedge block. Since the acoustic impedance of the wedge block is much smaller than that of the piezoelectric film, a significant portion of the acoustic energy will be reflected back without any matching layer. Hence, the acoustic impedance mismatch between the piezoelectric film and the wedge block can be improved by adopting the matching layer. Generally, the optimal acoustic impedance of the matching layer can be calculated by the following expression [39]:

(1) $$\;Z_{m}=\sqrt{Z_{p}Z_{w}}$$

where $Z_{m}$, $Z_{p}$ and $Z_{w}$ are the acoustic impedances of the acoustic impedance matching layer, piezoelectric film linear array and the wedge block, respectively. The wedge block is made of the Rexolite material because of its very low sound attenuation (0.3 dB/mm) and acoustic impedance (2.5 MRayl). Thus, we can obtain that $Z_{p}=30$ MRayl for the PZT-5H material and $Z_{w}=2.5$ MRayl for the Rexolite material, both of which have been used in the probe design. According to Formula (1), it can be calculated that the ideal acoustic impedance of the matching layer is 8.66 MRayl. Here, we use the custom tungsten carbide/epoxy resin composite (Guangdong Shantou Goworld Co., Ltd. Ultrasonic Instrument, Shantou, China) with an acoustic impedance of about 8.3 MRayl, which is intermediate between those of the PZT-5H and Rexolite layers and beneficial to achieve good acoustic impedance matching.

### 2.1. Angle of Wedge Block

**Figure 2:**
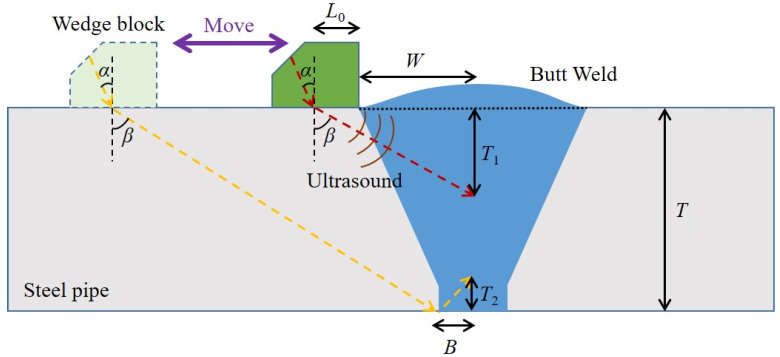
Detection of the butted weld with the primary and secondary ultrasound waves.

The wedge block is used to convert the longitudinal waves into shear waves after the ultrasound is transmitted from the wedge block into the steel pipeline. Considering two special cases that the butted weld area is inspected with primary and secondary ultrasound waves, respectively, as shown in Figure 2, it can be seen that the minimum values of the distance *T*_1_ and distance *T*_2_ can be expressed as:

(2) $$\;T_{1}=\frac{W+L_{0}}{\tan\left(\beta \right)}$$

(3) $$\;T_{2}=\frac{B}{\tan\left(\beta \right)}$$

where the width *W* of the single V-groove weld is generally between 0.5*T* and *T* (*T* is the thickness of the steel pipe), the length *L*_0_ is about 10 mm for our proposed PMUT array probe, and the width *B* can be ignored.

Because the sum of the two distances *T*_1_ and *T*_2_ should be no greater than the thickness (*T*) of the pipeline, we can obtain:

(4) $$\tan\left(\beta \right)⩾\frac{W+B+L_{0}}{T}$$

When the thickness of the steel pipeline varies from 10 to 30 mm, the calculated minimum refraction angle β will decrease from 56.3° to 39.8°. Conversely, too large β will inevitably increase the scanning distance and reduce the detection sensitivity. Therefore, a compromised refraction angle β of 55° has been adopted in order to effectively and accurately detect the weld defects in the steel pipelines with a thickness of no less than 10 mm. To obtain a refraction angle *β* of 55°, the incident angle *α* can be calculated according to the refraction principle:

(5) $$\;\frac{\sin\alpha }{C_{1}}=\frac{\sin\beta }{C_{2}}$$

where $C_{1}$ is the longitudinal sound velocity of the incident ultrasonic wave in the wedge block, and $C_{2}$ is the shear sound velocity of the refracted ultrasonic wave in the steel pipeline. Considering that $C_{1}$ is 2337 m/s for the Rexolite wedge block and $C_{2}\;$ is 3260 m/s for the steel pipeline, we can finally obtain the incident angle α of 36.0°, which is same as the angle of the wedge block in our probe design.

### 2.2. Probe Operation Frequency

Detection resolution and detection depth are two important parameters to determine the defect detection performance of ultrasonic transducer probes, which largely depends on the half-wavelength (*λ*/2) of the ultrasonic waves [40]. Generally, the wavelength of the ultrasound in the transmission medium is determined by the operation frequency (*f*) and the ultrasound velocity (*v*). The higher the operation frequency, the higher the detection resolution and the smaller the detection depth. In the case of the steel pipelines with the pipe wall thickness of 6~25 mm, in order to effectively identify the submillimeter defects, the recommended operation frequency of the ultrasonic transducer probe is 4~5 MHz when the speed of the ultrasound is assumed to be 5900 m/s. Therefore, the center frequency of the PMUT linear array probe is designed at 5 MHz. The resonance frequency of the probe at the TE vibration mode can be expressed by:

(6) $$f_{r}=\frac{1}{2t}\sqrt{\frac{c_{33}^{E}}{\rho }\frac{\left(1-\sigma^{E}\right)}{\left(1+\sigma^{E}\right)\left(1-\sigma^{E}\right)}}$$

where *t*, $c_{33}^{E}$, ρ, $\sigma^{E}$ and $k_{t}$ are the thickness, elastic stiffness constant, density, Poisson’s ratio and electromechanical coupling factor of the piezoelectric film. According to the expression (6), the thickness of the piezoelectric film can be calculated and is set to 0.40 mm for targeting an operation frequency of 5 MHz.

### 2.3. The Number of Array Elements

The number of elements in the PMUT array is an important factor, which will not only determine the directivity and width of the main lobe, but also affect the distribution and amplitude of the grating lobes. The goal of optimizing the PMUT array probe is to reduce the width of the main lobe, eliminate the distribution of the grating lobes and reduce their amplitude. The width of the main lobe is determined by two zeros near the deflection angle, and the position of the zero point is independent of the array width. When the array width (w) and the wavelength (λ)  of the ultrasonic waves in the transmission medium satisfy the criteria: w/λ≪1, the directivity function H(θ) of a linear array can be written as:

(7) $$H\left(\theta \right)=\left|\frac{\sin\left[\frac{\pi Nd\;\left(\sin\theta_{s}-\;\sin\theta \right)}{\lambda }\right]}{N\sin\left(\frac{\pi d\;\left(\sin\theta_{s}-\;\sin\theta \right)}{\lambda }\right)}\right|$$

where $\theta_{s}$ is the steering angle, *d* is the array pitch, *N* is the number of array elements.

**Figure 3:**
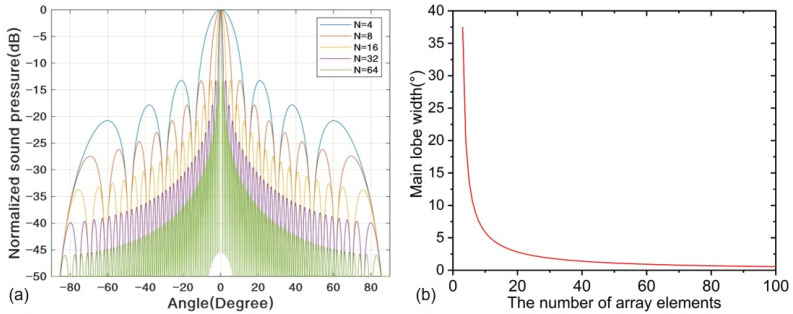
The influence of the number of array elements: (**a**) directivity and (**b**) the main lobe width.

When the azimuth angle is 0° and there is no steering, the directivity of the PMUT array with a different number of array elements can be plotted, as shown in Figure 3a. Let *H*(θ)=0, then there are:

(8) $$\frac{\pi d\left(\sin\theta_{s}-\sin\theta \right)N}{\lambda }=m\pi \left(m\neq 0,m\neq N\pi ,m\in Z\right)$$

According to Expression (8), its analytical result can be derived as: $\theta =\sin^{-1}\left(\sin\theta_{s}-m\lambda /Nd\right)$. The two angles corresponding to *m* = 1 and *m* = −1 represent the left and right zeros of the main lobe ($\theta_{s}>0$), and then, the main lobe width can be normalized as:

(9) $$\theta_{e}=\frac{1}{\pi }\left\{\sin^{-1}\left(\sin\theta_{s}+\frac{\lambda }{Nd}\right)-\sin^{-1}\left(\sin\theta_{s}-\frac{\lambda }{Nd}\right)\right\}$$

As can be seen from Equation (9), the smaller the steering angle, the narrower the main lobe; the larger the proportional expression d/λ, the narrower the main lobe; the greater the number of array elements, the narrower the main lobe. The relationship between the main lobe width $\theta_{e}$ and number of array elements *N* is plotted in Figure 3b. Furthermore, too many array elements will greatly increase the production cost, size and complexity of the signal line connection and back-end processing. Therefore, to strike a balance between these two concerns, a 16-element PMUT linear array is finally chosen.

### 2.4. Array Pitch

The grating lobes and side lobes that appear on both sides of the main lobe in the directivity chart of the PMUT array are unbeneficial to improve the detection resolution and accuracy, resulting in defect artifacts. In addition to the operation frequency and the element number of the PMUT array, the array pitch is another important parameter that can seriously affect the distribution and amplitude of grating lobes and side lobes. The location of their occurrence can be obtained by finding out the maximum directivity angle. For H(θ)=1, there are:

(10) $$\frac{\pi d\left(\sin\theta_{s}-\sin\theta \right)}{\lambda }=m^{\prime }\pi \left(m^{\prime }\in Z\right)$$

where $m^{\prime }=1$ corresponds to the first grating lobe, given by:

(11) $$\theta_{g1}=\sin^{-1}\left(\sin\theta_{s}-\frac{\lambda }{d}\right)$$

Let $\sin\theta_{s}-\lambda /d<-1$ to eliminate the first grating lobe and obtain the critical array pitch ($d_{cr}$):

(12) $$\;d_{cr}=\frac{\lambda }{1+\sin\theta_{s}}$$

When the array pitch is slightly less than $d_{cr}$, the grating lobes will still appear at $-90^{{}^{\circ}}$. Considering that there should be (N−2) side lobes between the main lobe and the grating lobe, the maximum pitch ($d_{max}$) that can eliminate the grating lobe is:

(13) $$\;d_{\max}\;=\;\frac{\lambda }{1+\sin\theta_{s}}\;\frac{N-1}{N}$$

As can be seen from Equation (13), $\lambda /2<d_{max}<\lambda$. When the pitch of the PMUT array elements is less than λ/2, no grating flaps are generated. Therefore, the pitch of the 16-element PMUT linear array is designed to be 0.5 mm, and the width of the piezoelectric film is set to 0.41 mm.

## 3. Electrical Impedance and Ultrasonic Echo Test

**Figure 4:**
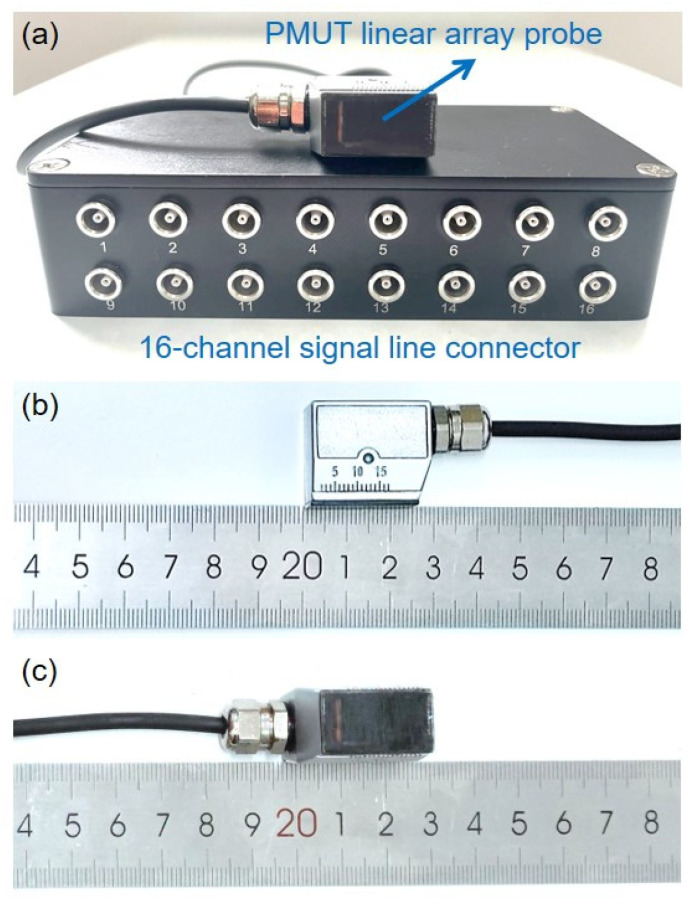
Pictures of the fabricated PMUT linear array probe: (**a**) PMUT probe with the connector, (**b**) lateral view of the PMUT probe, (**c**) bottom view of the PMUT probe.

**Figure 5:**
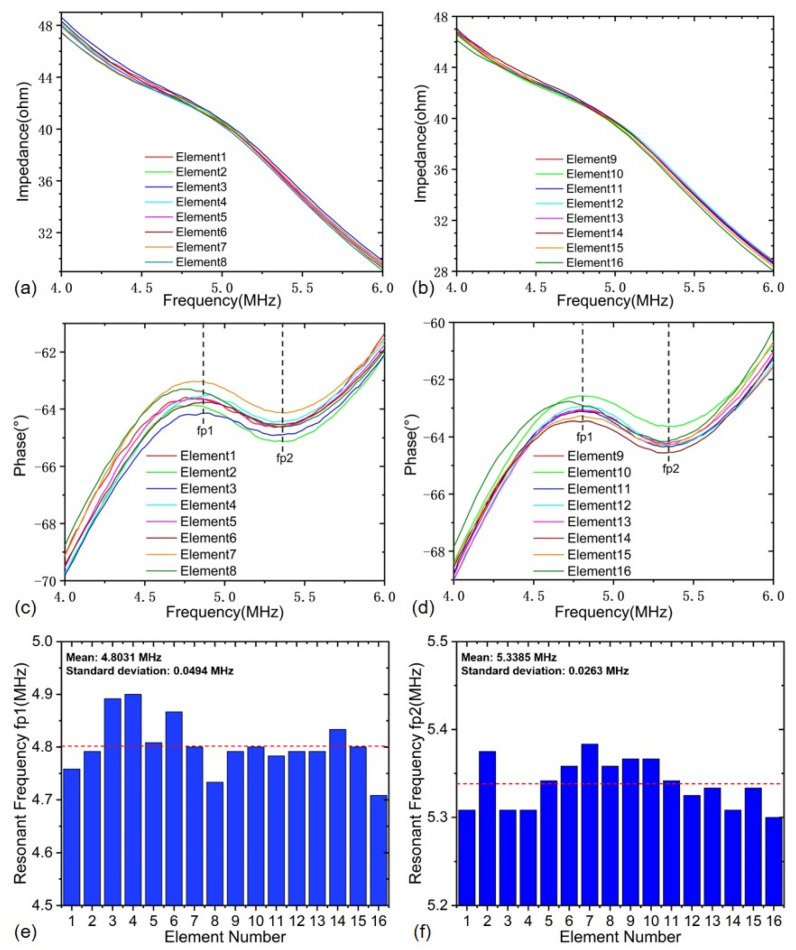
Impedance and phase spectrums of the developed PMUT linear array probe: (**a**) impedance curves for 1–8 elements, (**b**) impedance curves for 9–16 elements, (**c**) phase curves for 1–8 elements, (**d**) phase curves for 9–16 elements, (**e**) resonance frequency ($f_{p1}$) of each element, (**f**) resonance frequency ($f_{p2}$) of each element.

The proposed PMUT linear array probe has been fabricated and is shown in Figure 4, where a custom 16-channel signal line connector is used for signal transmission. The electrical impedance–frequency characteristic is necessary to analyze the electromechanical performance of the developed PMUT linear array probe. The impedance analyzer LCR-8110G (Goodwell Electronics Co., Ltd., Taipei City, China) was used to test the impedance and phase spectrum characteristics of the developed PMUT linear array probe. Since the wedge block is integrated inside the PMUT linear array probe, the wedge block will inevitably cause the frequency–impedance curve change of each PMUT element although all PMUT elements are theoretically designed to resonate at 5 MHz. Due to the damping effect of the wedge block, the impedance resonance characteristic curves become so flat that the resonance peak and anti-resonance peak are not apparent, as shown in Figure 5. However, it can be also seen from the frequency–phase characteristic curves that all 16 elements of the PMUT linear array probe in the frequency range of 4.0~6.0 MHz have very consistent resonance characteristics, where the average resonance frequency (*f*_*p*1_) is 4.8031 MHz and their standard deviation is 49.4 kHz. Compared with the theoretically designed frequency, the operation frequency deviation of the fabricated PMUT linear array probe is less than 5%, implying that the probe is processed successfully and meets the design requirements.

**Figure 6:**
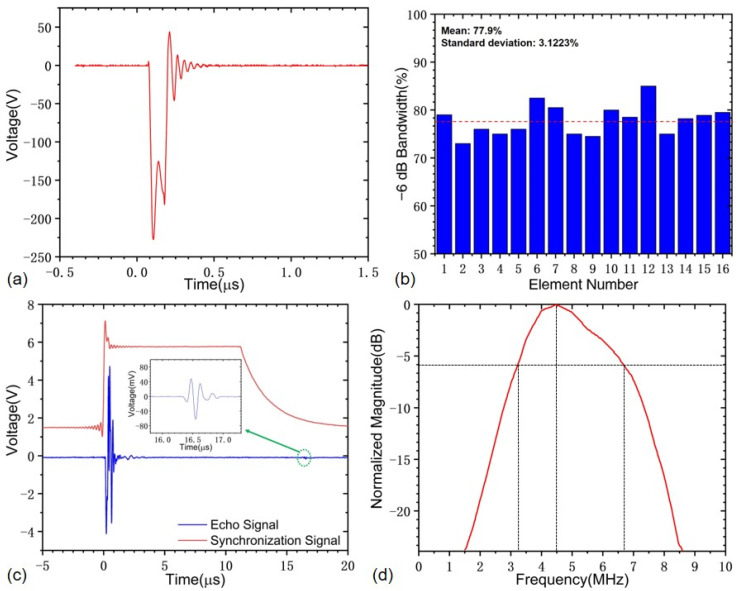
Ultrasonic characterization of the developed PMUT linear array probe: (**a**) excitation pulse, (**b**) −6 dB bandwidth of all 16 elements, (**c**) the transmitted synchronization signal and the received echo signal, (**d**) FFT spectrum.

In order to further analyze the ultrasonic characteristics of the developed PMUT linear array probe, the ultrasonic pulse echo testing was carried out using the CTS-8077PR ultrasonic pulser-receiver (Guangdong Shantou Goworld Co., Ltd. Ultrasonic Instrument). In the test, the electrical damping value was set to 80 Ω, the rectangular excitation pulse voltage was 150 V as shown in Figure 6a, the receiving gain was 0 dB, the pulse repetitive frequency was 1 kHz, the low-pass and high-pass filters were 10 MHz/1 MHz, and the Rexolite test block with a thickness of 20 mm was used. The transmitted synchronization signal was used to record the starting time during the testing. All elements have been tested, and their average −6 dB bandwidth is 77.9%, with a standard deviation of 3.1%, as shown in Figure 6b, indicating that the wide bandwidth design of the PMUT linear array probe can be realized after considerations of the acoustic impedance matching layer and the wedge block. Considering that the performance of the element No. 14 is closest to the average behaviors of all 16 elements, this element is taken as one typical element to illustrate the experimental results. It is noted that this is a necessary and reasonable simplification to avoid taking up too much space with a large number of similar figures. The experimental results of the element No. 14 are shown in Figure 6c. It can be concluded that the time-of-flight (ToF) of the transmitted ultrasound and the average duration of the ultrasonic echoes are about 16.4 and 0.2 μs, respectively. The peak-to-peak voltage of the ultrasonic echoes is 63 mV. The ultrasonic echo waveforms in the time domain are performed with the fast Fourier transforms (FFT) method, and the normalized amplitudes of the different frequency bands are plotted, as shown in Figure 6d, with the −6 dB bandwidth of 77.9%.

**Figure 7:**
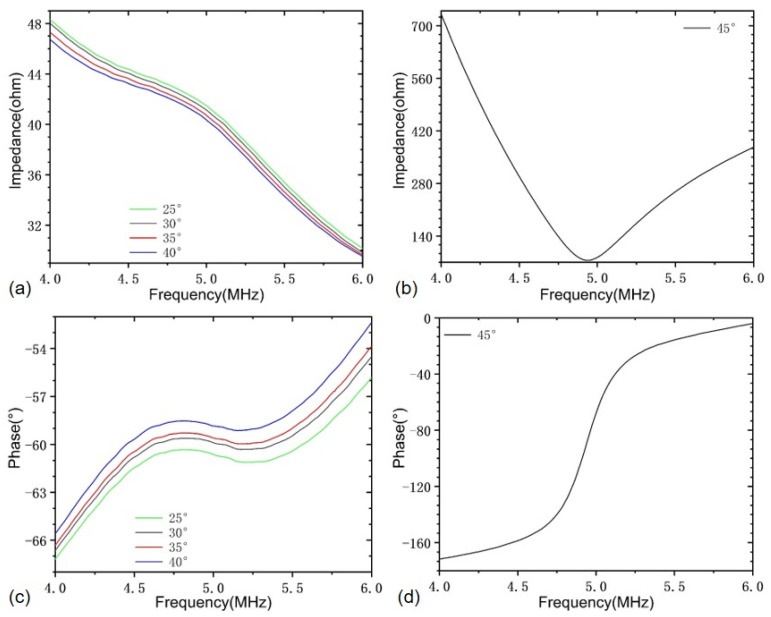
Impedance and phase spectra at different temperatures: (**a**,**b**) impedance and phase spectra in the temperature range from 25 to 40 °C; (**c**,**d**) impedance and phase spectra at 45 °C.

Furthermore, the thermal endurance property of the developed PMUT array probe device is also characterized. Considering that the piezoelectric film in the PMUT array device is made of PZT-5H material, whose Curie temperature is about 200 °C, a high temperature above 70 °C will cause a decrease in piezoelectricity and may result in the piezoelectric crystalline phase change. Thus, the temperature tests are performed in the temperature range of 25 to 45 °C, which covers most of the actual in situ testing conditions. The measured impedance and phase spectra of element No.1 as an example are shown in Figure 7. It can be concluded that all 16 PMUT elements have consistent resonance characteristics and exhibit good stability when the temperature is below 45 °C, with a frequency deviation of no larger than 2.5%. When the temperature increases to 45 °C, the impedance and phase characteristic will change dramatically, and such a change can be reversible if the temperature does not continue to increase or is kept too long. Nevertheless, the developed PMUT array probe can meet the test requirement at room temperature.

## 4. Weld Testing Experiment Results and Discussion

**Figure 8:**
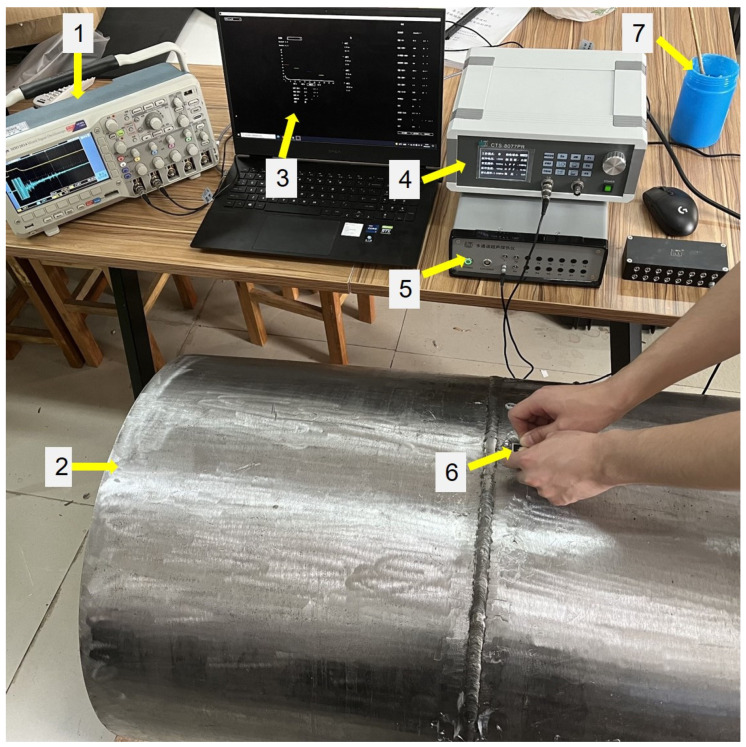
Experimental platform: 1—oscilloscope, 2—butt-welded pipeline test sample, 3—ultrasonic signal acquisition system interface, 4—ultrasonic pulser/receiver, 5—two-channel ultrasonic system, 6—PMUT linear array probe, 7—ultrasonic couplant.

The experimental platform for testing the PMUT linear array probe has been established, including the CTS-8077PR ultrasonic pulser-receiver, the CTS-02UT two-channel ultrasonic system (Guangdong Shantou Goworld Co., Ltd. Ultrasonic Instrument), the personal computer (PC) with a professional ultrasonic signal acquisition system software, MSO 2014 mixed signal oscilloscope (Tektronix, Inc., Beaverton, OR, USA), the butt-welded pipeline test sample and the ultrasonic couplant (Hydraulicil32 machine oil produced by Sri Lanka Petroleum (Chongqing) Co., Ltd., Chongqing, China), as shown in Figure 8, where the two-channel ultrasonic system can realize the excitation of the high-voltage (up to 300 V_pp_) pluses and the reception of the ultrasonic echo signals with an adjustable gain.

**Figure 9:**
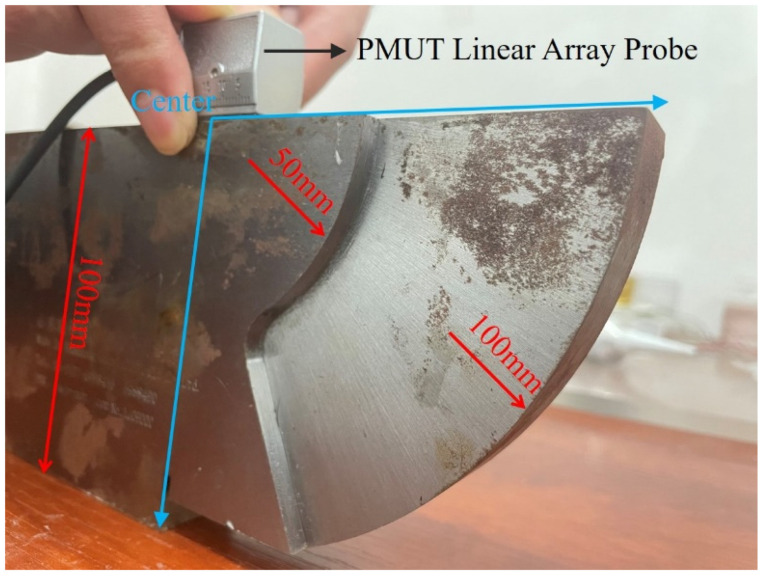
Illustration of calibration with the ultrasonic calibration test block.

**Figure 10:**
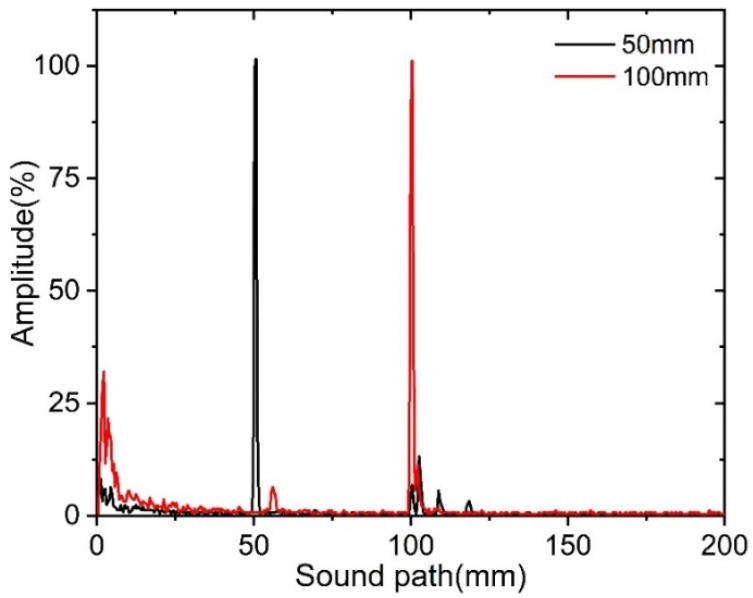
The calibrated ultrasonic echo signals.

The two-channel ultrasonic system was first calibrated before testing the designed PMUT linear array probe. The ultrasonic calibration test block is made of steel and consists of two concentric quarter circles, whose radii are 100 and 50 mm, respectively, as shown in Figure 9. During the calibration, the PMUT linear array probe operates at the self-transmitting and self-receiving modes. The PMUT probe is placed on the upper face of the ultrasonic calibration test block. When the PMUT probe faces the 50 mm circular arc surface, the maximum ultrasonic echo can be found by moving the probe back and forth. After that, the PMUT probe is changed to face the 100 mm circular arc surface, and then, the corresponding maximum ultrasonic echo can also be found by moving the probe back and forth, as shown in Figure 10. It can be seen that both ultrasonic echoes reflected from the 50 and 100 mm circular arc surfaces can accurately locate these two circular arc surfaces.

**Figure 11:**
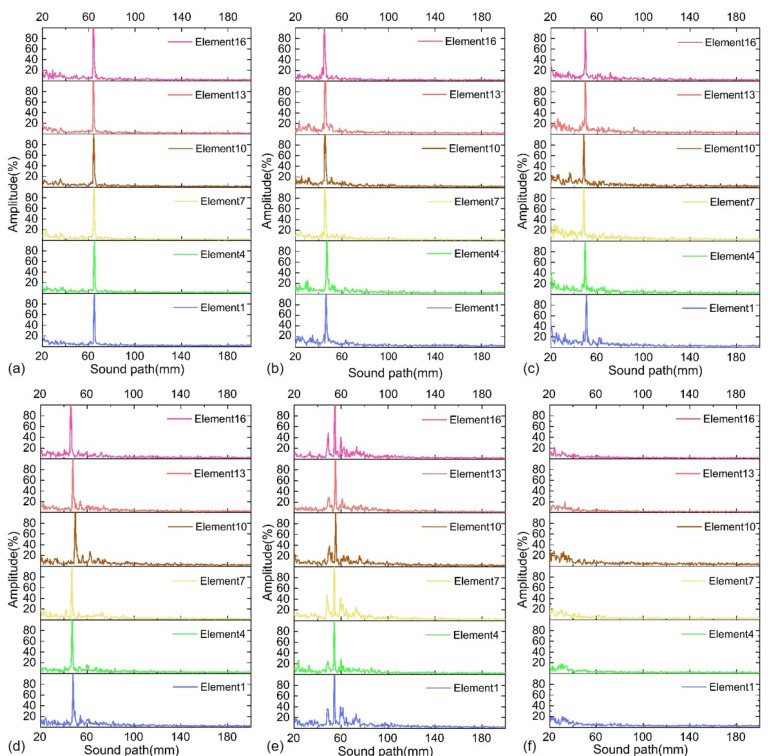
Detected location distributions for five different girth weld defects and defect-free case: (**a**) gas pores, (**b**) incomplete penetration, (**c**) cracks, (**d**) incomplete fusion, (**e**) slag inclusion, (**f**) no defect.

**Figure 12:**
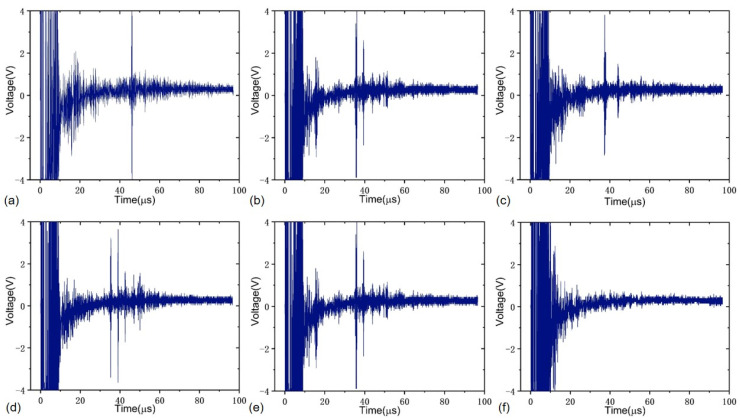
Received original ultrasonic echo signals for five different girth weld defects and defect-free case: (**a**) gas pores, (**b**) incomplete penetration, (**c**) cracks, (**d**) incomplete fusion, (**e**) slag inclusion, (**f**) no defect.

The butt-welded pipeline test sample is made of steel, and its length is 1000 mm. The outer diameter and pipe wall thickness of the pipeline test sample are 610 and 10 mm, respectively. The pipeline test sample contains five common kinds of girth butt weld defects with different depths and sizes, which are gas pores, incomplete penetration, cracks, incomplete fusion and slag inclusion. There are two samples for each kind of girth butt weld defect, and their structural sizes are listed in Table 1, where the minimum feature size of all defects is less than 500 μm, which has been checked through the X-ray testing. Both the butt-welded pipeline test sample and its X-ray testing report are provided by Dandong BaiHuiDa Testing Equipment Co., Ltd., Liaoning, China. It is noted that the ultrasound reflections on the contact face and side walls of the pipeline test sample are unavoidable during the ultrasonic inspection, which will produce some disturbance signals as artifacts. To minimize the interference of reflected echoes, the distance from the girth weld boundary to the center of the PMUT linear array probe was controlled at 20 mm. Furthermore, all five kinds of weld defects were tested with all PMUT array elements by using a CTS-02UT two-channel ultrasonic system. Here, the detected location distributions for five kinds of girth weld defects and defect-free case with some typical PMUT elements #1, #4, #7, #10, #13 and #16 are shown in Figure 11. It is noted that this is a necessary and reasonable simplification to avoid taking up too much space with a large number of similar figures. It can be seen from the results that all PMUT array elements are able to effectively detect the above five kinds of defects with a high consistency. For completeness, the received original ultrasonic echoes for both the five different defects and defect-free cases are recorded with the CTS-8077PR ultrasonic pulser-receiver, as shown in Figure 12. These results are highly consistent with the location distribution data, which demonstrate that the developed multi-channel PMUT linear array probe is effective and can be applied to accurately detect common weld defects of oil and gas pipelines.

**Figure 13:**
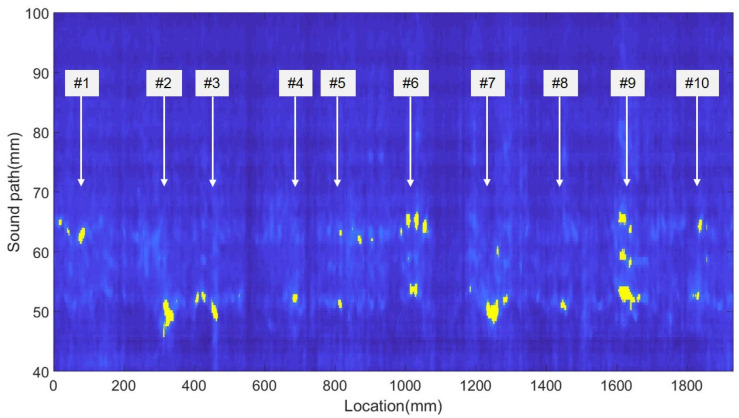
Internal defect detection signal map of the welding test block.

In order to further characterize the on-site inspection performance of the 16-channel PMUT linear array probe, the whole girth weld region of the butt-welded pipeline test sample was tested at a 2 mm interval with one element of the PMUT linear array probe. The distance between the probe and the edge of the girth weld was kept at 20 mm. The ultrasonic echoes were acquired and recorded with the CTS-8077PR ultrasonic pulser-receiver (Guangdong Shantou Goworld Co., Ltd. Ultrasonic Instrument). Then, the collected ultrasonic echo data at different test points were evaluated with the imaging processing method by MATLAB software. The results are shown in Figure 13. It can be seen that the abnormal signals (marked in yellow color) appear at the special locations where 10 weld defects in five different types have been artificially created. As a contrast, the strength of the ultrasonic echoes in the locations without the artificial defects are comparable to the background reference signals (marked in blue color).

## 5. Conclusions

In this study, a novel 16-channel broadband piezoelectric micro ultrasonic transducer (PMUT) linear array probe is theoretically designed and fabricated for detecting the butt-welded defects in pipelines. Mathematical modeling has been made to optimize the structural parameters of the PMUT array probe. After comprehensive electrical impedance and phase testing, the average resonance frequency and standard deviation of all 16 designed PMUT elements are 4.8031 and 49.4 kHz, offering a maximum operating frequency deviation of less than 5%. The frequency responses indicate that the theoretical model can effectively guide the design of the piezoelectric ultrasonic transducer array. The ultrasonic pulse-echo performance tests demonstrate that the −6 dB bandwidth of the PMUT array probe can be up to 77.9%, which is larger than the data of most previously reported piezoelectric ultrasonic transducers, especially PMUTs. In order to further verify its applicability in the industrial non-destructive testing, the developed PMUT linear array probe has been applied to accurately detect the standard ultrasonic calibration block with radii of 50 and 100 mm. Furthermore, five common internal defects (gas pores, incomplete penetration, cracks, incomplete fusion and slag inclusion) in pipeline girth butt welds with a minimum feature size of less than 500 μm have also been detected and observed with the fabricated PMUT array probe, exhibiting a high detection resolution. Benefited from its simple and miniature structure and advantageous behaviors, the developed PMUT array probe promisingly facilitates other structure health monitoring applications, in addition to modern medical imaging and therapy. There are also a few limitations in the NDT applications. The first one is that the PMUT array probe cannot work in high temperature environment, which is mainly attributed to the adoption of PZT-5H with low Curie temperature. Furthermore, the number of elements in the PMUT array may not be recommended to be more than 32 when there is a specific requirement for the volume of the array probe and the signal line connector matches with the probe. Future work will involve filed studies combined with the time of flight diffraction (TOFD) and phased-array methods to further demonstrate the effectiveness of the fabricated PMUT probe.

## Figures and Tables

**Table 1:** Structural sizes of girth weld defects in the pipeline test sample.

Number	Defect Category	Location (mm)	Depth (mm)	Length (mm)
#1	Gas pores	73	5	25
#2	Incomplete penetration	307	10	23
#3	cracks	458	surface	10
#4	Incomplete fusion	661	8	18
#5	Slag inclusion	848	7	24
#6	Gas pores	1041	5	14
#7	Incomplete penetration	1231	10	40
#8	cracks	1445	surface	30
#9	Incomplete fusion	1592	8	63
#10	Slag inclusion	1802	7	47

## Data Availability

Not applicable.
